# A Preliminary Analysis of Sex-Based Differences in Immune Status, ART Adherence, and Opportunistic Infections Among HIV-Positive Patients in Rural Eastern Cape, South Africa

**DOI:** 10.3390/tropicalmed11070183

**Published:** 2026-07-04

**Authors:** Ikhona Ntshobane, Dominic Targema Abaver

**Affiliations:** Department of Laboratory Medicine and Pathology, School of Medicine, Faculty of Medicine and Health Sciences, Walter Sisulu University, Mthatha 5100, Eastern Cape, South Africa; dabaver@wsu.ac.za

**Keywords:** HIV, opportunistic infections, candidiasis, CD4 count, viral load, ART adherence, immune suppression, South Africa

## Abstract

Background: Opportunistic infections remain a significant cause of morbidity among people living with HIV (PLHIV) in sub-Saharan Africa despite expanded access to antiretroviral therapy (ART). This study evaluated the association between immune status, viral load suppression, ART adherence, and the risk of opportunistic infections among HIV-positive patients receiving ART in the rural Eastern Cape, South Africa. Methods: A retrospective cross-sectional study was conducted using clinical records of HIV-positive patients attending an HIV clinic in Mthatha between January 2021 and December 2024. Demographic characteristics, CD4 counts, viral load results, ART regimens, adherence status, and documented opportunistic infections were extracted. Viral load suppression was defined according to WHO guidelines as <1000 copies/mL. CD4 counts were categorized as <200, 200–499, and ≥500 cells/mm^3^. Multivariable logistic regression analysis was performed to identify independent predictors of opportunistic infections. Results: A total of 155 patients (105 females, 50 males) were included. Females demonstrated significantly higher mean CD4 counts than males (*p* = 0.037) and better ART adherence (*p* < 0.001). Tuberculosis, hepatitis B virus, and herpes simplex virus infections were more prevalent among males, whereas candidiasis was significantly more common among females (*p* = 0.034). In multivariable analysis, CD4 count < 200 cells/mm^3^, unsuppressed viral load, and poor ART adherence were independently associated with increased odds of opportunistic infections. Conclusions: Immune suppression and suboptimal ART adherence significantly increase the risk of opportunistic infections among HIV-positive patients in rural South Africa. Strengthening adherence interventions and early immune monitoring may reduce infection burden in high-prevalence settings.

## 1. Introduction

Opportunistic infections (OIs) continue to contribute substantially to morbidity and mortality among people living with Human immunodeficiency virus (PLHIV) in sub-Saharan Africa, even in the context of expanded access to antiretroviral therapy (ART) [1,2]. In South Africa, an estimated 7.8 million individuals are living with HIV, with a substantial proportion presenting with advanced immunosuppression [3]. HIV infection leads to progressive depletion of CD4 T-lymphocytes, resulting in immune suppression. This immunocompromised state predisposes patients to a wide range of opportunistic infections (OIs), including tuberculosis (TB), herpes simplex virus (HSV) infections, hepatitis B virus (HBV), and mucosal candidiasis [4,5]. These infections are consequences of HIV-associated immune deficiency rather than primary causes of immune dysfunction. They not only worsen patient outcomes but also increase healthcare utilization, particularly in resource-limited settings where diagnostic and treatment capacities are constrained [6]. Despite national efforts to expand ART coverage, the Eastern Cape province remains burdened with high rates of unsuppressed viral loads and late-stage HIV disease [7]. Routine clinical markers, including CD4 count, viral load, ART regimen, adherence status, and comorbidities, are widely recognized predictors of OI risk and treatment outcomes [8]. However, locally derived evidence on how these markers relate to OI susceptibility remains limited. In many district-level facilities, clinicians rely on general risk profiles rather than context-specific epidemiological data, limiting their capacity to identify patients at elevated risk for OIs [9].

Mucosal candidiasis is a particularly relevant opportunistic infection in this context, as it remains common among PLHIV and is associated with immune suppression [10]. The increasing prevalence of azole-resistant non-albicans Candida species further complicates clinical management, especially in regions where access to newer antifungal agents is limited. Retrospective analyses of existing clinical records offer a feasible approach to evaluating immune status, ART adherence, and OI susceptibility, even in the absence of comprehensive microbiological data [11]. Such analyses can provide valuable insights into patient vulnerabilities, supporting targeted interventions to prevent OIs and improve outcomes.

The Eastern Cape has notable gaps in district-level evidence describing patterns of immune suppression, ART adherence, and OI occurrence. Understanding these patterns is essential for improving both individual patient care and health system planning. Evaluating gender differences in ART adherence, immune recovery, and comorbid infections may further refine risk-stratification strategies, as prior studies have shown that males and females often differ in immune responses, treatment adherence, and OI susceptibility [12,13].

This study aimed to describe the demographic and clinical characteristics of HIV-positive patients on ART in rural Eastern Cape and examine how CD4 counts, viral load suppression, and treatment adherence relate to the risk of OIs, including candidiasis. The analysis also explored gender-stratified patterns to identify subgroups at higher risk, providing actionable evidence for clinicians and program planners in high HIV-burden, resource-limited settings.

## 2. Materials and Methods

### 2.1. Study Design

This retrospective study employed a descriptive-analytical design. The descriptive component summarized patient demographic characteristics, clinical indicators, antiretroviral therapy (ART) regimens, adherence status, co-morbidities, and documented outcomes. The analytical component examined associations between immune status, treatment-related variables, and susceptibility to opportunistic infections, including candidiasis. The study workflow involved systematic screening of patient records, application of eligibility criteria, structured data extraction, data verification, statistical analysis, and interpretation of immune and treatment profiles.

### 2.2. Study Setting

The study was conducted at an HIV Clinic located in Mthatha, a major town in the OR Tambo District of the Eastern Cape Province, South Africa. Mthatha serves as a key service hub for surrounding rural communities and is characterised by a high burden of HIV infection, limited healthcare resources, and long travel distances to tertiary referral centres.

Mthatha is strategically positioned along major national transport routes connecting the Eastern Cape to other provinces, including the N2 corridor linking it to East London (Gqeberha/Port Elizabeth region) and Durban. The area is part of a predominantly rural and underserved region of the Eastern Cape, where access to specialised healthcare services is often constrained by geographic isolation and socioeconomic factors.

The HIV clinic in Mthatha provides comprehensive HIV care, including antiretroviral therapy (ART) initiation and follow-up, routine immunological and virological monitoring, and management of HIV-related co-morbidities. The clinic maintains detailed longitudinal patient records of comorbid conditions and clinical outcomes.

Given its central role in the regional HIV care cascade, the clinic serves a large and diverse patient population referred from surrounding peri-urban and rural health facilities. This makes it a suitable setting for retrospective analyses aimed at understanding disease patterns, treatment outcomes, and opportunistic infections in a real-world, resource-limited context.

### 2.3. Study Population, Eligibility Criteria, and Sampling Strategy

The study population comprised confirmed HIV-positive patients who attended the HIV Clinic in Mthatha between January 2021 and December 2024. Eligible records were required to include complete information on demographic characteristics (age and gender), CD4 count, viral load, ART regimen, adherence status, co-morbidities, and documented clinical outcomes. Records were excluded if HIV status was not confirmed or if key clinical, laboratory, or treatment information was incomplete.

A total population sampling approach was employed, whereby all available patient records within the study period were screened for eligibility, and only those meeting the inclusion criteria were included in the final dataset. Although a theoretical minimum sample size of 323 participants was estimated using the single population proportion formula at a 95% confidence level and a 5% margin of error, this calculation was used solely to assess sample adequacy. Due to the retrospective design and limited availability of complete records, 155 eligible patient records were included in the final analysis.

While the final sample size was smaller than the calculated minimum, it was considered adequate for descriptive and correlational analyses. However, the reduced sample size may have limited statistical power for subgroup and multivariable analyses, and this limitation has been acknowledged.

### 2.4. Data Collection Instrument and Procedure

Data were extracted from patient medical records using a structured data abstraction tool developed specifically for this study. The tool was designed to ensure uniform and systematic capture of relevant variables based on routinely documented clinical parameters in the HIV clinic records. A data dictionary was developed to standardise variable definitions, coding categories, and units of measurement (e.g., CD4 count in cells/mm^3^ and viral load in copies/mL).

Variables collected included demographic characteristics (age and gender), immunological markers (most recent CD4 count), virological markers (most recent viral load), ART-related information (current regimen and documented adherence status), recorded co-morbidities (including tuberculosis, hepatitis B, hypertension, and diabetes mellitus), documented opportunistic infections, and clinical follow-up outcomes. For patients with multiple laboratory results during the study period, the most recent CD4 count and viral load were recorded to reflect current immune and virological status.

Eligible records were identified from the clinic registry for the period January 2021 to December 2024 and screened against inclusion and exclusion criteria prior to data extraction. Records with missing key variables were excluded at the screening stage and not included in the final dataset.

The abstraction tool was piloted on approximately 10 randomly selected records to assess clarity, completeness, and consistency. Minor refinements were made to improve the standardisation of variable interpretation.

Data were manually extracted by the primary investigator and entered a pre-designed electronic database. To ensure accuracy and reliability, a second reviewer independently verified all entries against the original records, and discrepancies were resolved through consensus review.

ART adherence was assessed from routine clinical documentation and categorised as “good” or “poor” by the clinician. Objective adherence measures, such as pill counts or pharmacy refill records, were not available due to the study’s retrospective design. Opportunistic infections were identified from documented clinical diagnoses or laboratory-confirmed results; however, asymptomatic or undocumented infections may have been underreported due to reliance on routine clinical records.

### 2.5. Study Variables

The primary dependent variable was the presence of documented opportunistic infections. Secondary outcomes included candidiasis. Independent variables included CD4 count, viral load suppression status, ART adherence, age, sex, ART regimen, and comorbidities. These variables were analyzed to characterize immune status and treatment profiles and to explore their associations with opportunistic infections.

### 2.6. Statistical Analysis

Data were analysed using IBM SPSS Statistics version 23.0 (IBM Corp., Armonk, NY, USA). Continuous variables were assessed for normality using the Shapiro–Wilk test and presented as mean ± standard deviation (SD), while categorical variables were summarised as frequencies and percentages.

Comparisons between males and females were conducted using independent-samples *t*-tests for normally distributed continuous variables and chi-square or Fisher’s exact tests for categorical variables, as appropriate. Associations between ART adherence, immune status (CD4 count), viral load, and co-morbidities were further explored using sex-stratified analyses.

Correlation analyses were performed using Pearson’s correlation coefficient for normally distributed variables and Spearman’s rank correlation for non-normally distributed or ordinal variables. Correlation strength was interpreted according to Cohen’s guidelines (0.1 = small, 0.3 = moderate, 0.5 = strong).

Multivariable logistic regression analysis was conducted to identify independent predictors of opportunistic infections. Variables included in the model were selected based on clinical relevance and univariate analysis. To minimise overfitting, the number of predictor variables was restricted in line with events-per-variable (EPV) principles, ensuring model stability given the sample size.

### 2.7. Data Management

Patient data were anonymised prior to analysis by removing all personal identifiers, including names and hospital or folder numbers, and assigning unique study codes. Data were captured from clinical records and stored in a structured database, with access restricted to authorised members of the research team.

To ensure data quality and integrity, a double-entry system was implemented, with independent datasets cross-checked for discrepancies. Data cleaning procedures included verification of inconsistencies, outliers, and missing values against the original source documents. Where necessary, incomplete or ambiguous entries were resolved by consulting primary records, and variables with substantial missing data were handled appropriately during analysis.

Data security was maintained throughout the study by restricting access, using secure storage systems, and ensuring that no identifiable information was retained in the analytical dataset. All data handling procedures complied with institutional data protection policies and ethical guidelines.

Upon completion of the study, anonymised data will be securely archived for a defined period in accordance with institutional and regulatory requirements. Findings from the study will be disseminated through peer-reviewed publications, conference presentations, and stakeholder engagement with relevant health authorities to inform policy and clinical practice. No individual participant will be identifiable in any reports or publications arising from this study.

### 2.8. Ethical Considerations

This retrospective study utilised anonymised patient records and posed minimal risk to participants. Ethical approval was obtained from the Walter Sisulu University Health Sciences Research Ethics Committee (WSU-HREC 111/2025, 2 October 2025), the Eastern Cape Department of Health (ECDoH EC_202507_016, 11 July 2025), the OR Tambo District Department of Health (19 September 2025), and the King Sabatha Dalindyebo Local Municipality (19 September 2025).

Permission to access patient records was granted by the management of the Mthatha Gateway Clinic. The study was conducted in accordance with the principles of the Declaration of Helsinki and relevant national and institutional ethical guidelines.

## 3. Results

### 3.1. Demographic and Clinical Characteristics

A total of 155 HIV-positive patients receiving ART were included in the analysis (105 females, 50 males). The mean age was similar between females and males (40.57 ± 1.20 vs. 40.39 ± 1.47 years; *p* = 0.167). Table 1 presents the demographic and clinical characteristics of HIV-positive patients receiving ART in the Eastern Cape.

Females demonstrated higher mean CD4 counts than males (533.85 ± 26.82 vs. 399.80 ± 31.23 cells/mm^3^; *p* = 0.037). Viral load was lower in females, though the difference was not statistically significant (2263.44 ± 1188.58 vs. 3443.16 ± 1699.20 copies/mL; *p* = 0.329). Good ART adherence was more common among females (89.6% vs. 66%; *p* < 0.001).

Comorbid infections, such as TB, HBV, and HSV, were more prevalent in males (*p* = 0.034), Comorbid infections, such as TB, HBV, and HSV, were more prevalent in males, while vulvovaginal candidiasis was significantly more prevalent among females (*p* = 0.037).

### 3.2. ART Adherence and Immune Markers

Table 2 shows the correlation between ART adherence and immunological and virological markers stratified by sex. In females, adherence correlated negatively with CD4 decline (r = −0.435; *p* < 0.001) and positively with viral load suppression (r = 0.518; *p* < 0.001). In males, adherence showed a stronger negative correlation with CD4 decline (r = −0.691; *p* < 0.001) and a positive correlation with viral suppression (r = 0.401; *p* = 0.004).

### 3.3. Associations Among Immune Status, Viral Load, ART Adherence, and Comorbidities

Sex-stratified correlation analysis confirmed that stronger immune recovery (higher CD4 counts) was associated with lower viral load and better ART adherence. Table 3 presents the associations among immune status, viral load, ART adherence, and comorbidities.

### 3.4. Multivariable Logistic Regression

Table 4 presents the multivariable logistic regression analysis of predictors of opportunistic infections among HIV-positive patients receiving ART in rural Eastern Cape. Patients with CD4 counts below 200 cells/mm^3^ had significantly higher odds of opportunistic infections compared with those with higher CD4 counts (AOR = 3.82, 95% CI: 1.74–8.41; *p* = 0.001). Poor ART adherence was the strongest predictor of opportunistic infections (AOR = 4.67, 95% CI: 2.01–10.85; *p* < 0.001). Due to the sample size, sensitivity analyses were limited, and this has been acknowledged as a study limitation.

## 4. Discussion

This study characterised sex-based differences in immune status, ART adherence, and comorbid infections among HIV-positive patients receiving ART in a rural Eastern Cape setting. Three principal findings emerged. First, females demonstrated significantly higher CD4 counts and better ART adherence compared with males. Second, comorbid infections differed by sex, with tuberculosis (TB), hepatitis B virus (HBV), and herpes simplex virus (HSV) more prevalent among males, while vulvovaginal candidiasis was significantly more prevalent among females (41%), whereas genital candidiasis presenting as penile infection occurred in only 2% of males. Third, ART adherence was strongly associated with immune recovery and viral suppression in both sexes, with stronger associations observed in males.

The higher CD4 counts observed among females may reflect sex-related immunological differences in HIV pathogenesis and immune reconstitution under ART. Women generally exhibit stronger innate and adaptive immune activation driven by oestrogen-mediated enhancement of CD4+ T-cell responses and higher baseline immune activation, which can result in higher CD4 cell counts during treated HIV infection [14,15]. Conversely, males are more likely to present with advanced immunosuppression due to delayed engagement in care, which limits optimal CD4 recovery even after ART initiation. This delayed presentation may also contribute to a more pronounced immunological “reconstitution gap” in males, where viral suppression does not translate into equivalent CD4 restoration.

The observed association between ART adherence and immune recovery is consistent with established HIV treatment dynamics, in which sustained viral suppression reduces immune activation and enables progressive CD4+ T-cell reconstitution. Poor adherence in males may therefore perpetuate intermittent viraemia, immune activation, and incomplete CD4 recovery, thereby increasing susceptibility to opportunistic infections such as TB, HBV, and HSV. In contrast, better adherence among females likely facilitates more stable viral suppression and improved immune reconstitution kinetics, contributing to higher CD4 counts over time.

Sex differences in comorbid infections further reflect underlying immunological and behavioural factors. The higher burden of TB, HBV, and HSV among males may be linked to both advanced immunosuppression at presentation and reduced healthcare engagement, while the predominance of candidiasis among females likely reflects local mucosal immune vulnerability in the context of HIV-associated CD4 depletion. These sex-specific patterns highlight the interplay between immune recovery dynamics, pathogen susceptibility, and behavioural determinants of treatment outcomes.

Collectively, these findings reinforce that sex-based differences in HIV outcomes are not solely behavioural but are also shaped by biological differences in immune regulation and ART-driven immune reconstitution. Targeted interventions that address both adherence behaviour and late presentation in males are therefore essential to optimise immunological recovery and reduce opportunistic infection burden in rural HIV programmes [16,17].

The lack of a statistically significant difference in viral load between sexes, despite differences in CD4 count and adherence, may reflect variability in treatment duration or regimen potency. Nevertheless, the direction of association—lower viral load in females—remains consistent with large multicentre analyses showing improved virological outcomes among women on ART [18].

The higher prevalence of TB, HBV, and HSV among males is consistent with epidemiological data from Southern Africa, where TB-HIV co-infection rates are disproportionately higher in men [19]. HBV co-infection remains a recognised challenge in HIV programmes across Africa, influencing disease progression and liver-related morbidity [20]. Conversely, the markedly higher prevalence of candidiasis among females likely reflects biological susceptibility, particularly vulvovaginal candidiasis, which remains one of the most common opportunistic infections in women living with HIV globally [21]. These sex-specific infection patterns underscore the importance of integrated screening within ART programmes.

Correlation analyses confirmed strong associations between ART adherence and both immunological and virological markers. Better adherence was associated with higher CD4 counts and lower viral load, consistent with established evidence that sustained ART adherence is the strongest predictor of immune reconstitution and viral suppression [22]. The stronger association between adherence and immunological improvement among males likely reflects differences in healthcare engagement and timing of ART initiation rather than inherent biological differences. Similar patterns have been observed in longitudinal African cohorts, where improvements in adherence yielded substantial immune recovery among previously immunocompromised male patients [23].

The inverse association between ART adherence and comorbid infections further supports the protective role of sustained viral suppression in reducing the risk of opportunistic infections. Global data consistently demonstrate that improved ART adherence reduces the incidence of OIs, including TB and candidiasis [24]. Our sex-stratified findings contribute localised evidence from a rural South African context, where healthcare access disparities may exacerbate infection risk. Overall, the observed sex differences should be interpreted with caution, as they are likely influenced by behavioural and healthcare engagement factors, particularly ART adherence, rather than sex as a direct biological determinant of treatment response.

### Public Health Relevance, Significance, and Implications

These findings have important public health relevance in the context of sub-Saharan Africa, where HIV remains a leading cause of morbidity and mortality. Rural provinces such as the Eastern Cape face structural healthcare challenges, including limited access to specialized services, workforce shortages, and socioeconomic barriers to care. Sex disparities in adherence and immune recovery, as observed in this study, directly affect progress toward epidemic control and sustainable health system strengthening.

The demonstrated association between adherence and both immune and infectious outcomes highlights adherence as a critical public health lever. In high-burden settings, even modest improvements in ART adherence can substantially reduce opportunistic infections, decrease hospitalizations, and lower healthcare costs. The disproportionate burden of TB and HBV among males also has broader transmission implications, as untreated or poorly controlled co-infections contribute to ongoing community spread. Addressing these disparities is therefore essential not only for individual patient outcomes but also for population-level disease control.

From a policy perspective, the results support the implementation of differentiated service delivery models that specifically target male engagement in HIV care. Community-based adherence support, workplace testing initiatives, and flexible clinic hours may improve retention among men. Strengthened integration of TB and HBV screening within ART programs is also warranted, particularly for male patients.

For female patients, routine screening and early treatment of candidiasis and other opportunistic infections should remain integral to comprehensive HIV care. Moreover, the strong correlations observed between adherence and virological suppression emphasize the need for continuous adherence monitoring systems, including digital adherence tools and community health worker support in rural settings.

At the health systems level, these findings reinforce the importance of sustained viral load monitoring and timely immunological assessment to prevent opportunistic infections. Enhancing data quality within routine clinical records, as demonstrated in this study, can further strengthen surveillance and inform targeted interventions.

Cryptococcosis is a known opportunistic infection among HIV-infected individuals in sub-Saharan Africa, particularly in patients with advanced immunosuppression. However, cryptococcal infection was not included in the present dataset, as no recorded cases or screening data were available in the reviewed patient records. This highlights the need for improved surveillance and comprehensive recording of opportunistic infections in rural healthcare settings.

## 5. Limitations

This study has several limitations that should be considered when interpreting the findings. First, the retrospective design limits causal inference and relies on routine clinical documentation, which may introduce reporting bias, particularly in assessing ART adherence. Second, the study depended on available patient records, which limited the inclusion of some important opportunistic infections, such as cryptococcosis, as no screening or recorded cases were available in the dataset.

Third, some clinical and laboratory variables were not consistently documented, affecting the completeness of the analysis. The absence of data on several clinically relevant variables, including parasitic infections, CMV and HCV co-infections, as well as BMI and nutritional status, may have introduced residual confounding, as these factors can influence immune status and susceptibility to opportunistic infections.

In addition, the relatively modest sample size, particularly in subgroup analyses, may have reduced statistical power. The single rural study setting may also limit the generalisability of the findings to other regions.

Nevertheless, the use of verified patient records provides valuable real-world evidence from a rural, high HIV-burden context that remains underrepresented in the literature. Future studies should incorporate broader clinical variables and multi-site designs to provide a more comprehensive assessment of patient risk profiles.

## 6. Conclusions

Sex-based differences in immune recovery, ART adherence, and comorbidity burden remain evident among HIV-positive patients in the Eastern Cape. Poor ART adherence, low CD4 count, and unsuppressed viral load were key predictors of opportunistic infections. Targeted adherence interventions, particularly among male patients, and integrated screening for comorbid infections are essential to improve clinical outcomes. Strengthening rural HIV care systems will be critical for sustained epidemic control in high-burden settings.

## Figures and Tables

**Table 1:** Demographic and clinical characteristics of HIV-positive patients by sex in the Eastern Cape.

Variable	Female	Male	*p*-Value
**CD4 count (cells/mm^3^)**	533.85 ± 26.82	399.80 ± 31.23	0.037
**Viral load (copies/mL)**	2263.44 ± 1188.58	3443.16 ± 1699.20	0.329
**Age (years)**	40.57 ± 1.20	40.39 ± 1.47	0.167
**ART adherence (good)**	93 (89.6%)	33 (66%)	<0.001
**TB positive**	3 (2.86%)	3 (6%)	0.041
**HBV positive**	21 (20%)	4 (8%)	0.029
**HSV positive**	0 (0%)	3 (6%)	0.012
**Candidiasis positive**	(V Candidiasis) 43 (41%)	(Penile candidiasis) 1 (2%)	0.037

Abbreviations: ART, antiretroviral therapy; TB, tuberculosis; HBV, hepatitis B virus; HSV, herpes simplex virus; V Candidiasis, vulvovaginal candidiasis.

**Table 2:** Correlation between ART adherence and immune markers, stratified by sex.

Gender	Variable	r	*p*-Value
**Female**	CD4 count	−0.435	<0.001
**Female**	Viral load	0.518	<0.001
**Male**	CD4 count	−0.691	<0.001
**Male**	Viral load	0.401	0.004

**Table 3:** Associations among immune status, viral load, ART adherence, and comorbidities.

Sex	Variables	r	*p*-Value
**Female**	CD4 vs. Viral load	−0.26	0.007
**Female**	CD4 vs. ART adherence	−0.44	<0.001
**Female**	Viral load vs. ART adherence	0.52	<0.001
**Female**	ART adherence vs. Comorbidities	−0.72	<0.001
**Male**	CD4 vs. Viral load	−0.37	0.009
**Male**	CD4 vs. ART adherence	−0.69	<0.001
**Male**	Viral load vs. Comorbidities	−0.63	<0.001
**Male**	ART adherence vs. Comorbidities	−0.29	0.05

Abbreviations: ART, antiretroviral therapy. *p* < 0.05 (male); *p* < 0.01 (female).

**Table 4:** Multivariable logistic regression analysis of predictors of opportunistic infections among HIV-positive patients receiving ART in rural Eastern Cape.

Variable	Adjusted Odds Ratio (AOR)	95% Confidence Interval	*p*-Value
**CD4 count < 200 cells/mm^3^**	3.82	1.74–8.41	0.001
**Viral load ≥ 1000 copies/mL**	2.91	1.36–6.22	0.006
**Poor ART adherence**	4.67	2.01–10.85	<0.001
**Male sex**	1.58	0.73–3.41	0.243
**Age ≥ 40 years**	1.21	0.58–2.53	0.611

Abbreviations: AOR, adjusted odds ratio; CI, confidence interval; ART, antiretroviral therapy.

## Data Availability

Data is available from the corresponding author upon reasonable request.

## Abbreviations

The following abbreviations are used in this manuscript:

ART	Antiretroviral therapy
CD4	Cluster of differentiation 4
HBV	Hepatitis B virus
HIV	Human immunodeficiency virus
HSV	Herpes simplex virus
OI	Opportunistic infection
PLHIV	People living with HIV
TB	Tuberculosis
WHO	World Health Organization
